# Case Report: Case report: Mixed infection of *Plasmodium vivax *and *Plasmodium falciparum *in a tertiary hospital

**DOI:** 10.12688/f1000research.53162.1

**Published:** 2021-08-09

**Authors:** Abeer M. Al-Subaie

**Affiliations:** 1 Department of Clinical Laboratory Sciences, College of Applied Medical Sciences, Imam AbdulRahman Bin Faisal University, Dammam, Saudi Arabia

**Keywords:** Malaria, Falciparum malaria, Plasmodium vivax, Plasmodium falciparum, case report

## Abstract

Mixed infections with two or more species of *Plasmodium *are frequently reported due to vector factors, parasite factors (formation of hypnozoites) and host factors (residing in endemic areas, travel to endemic areas, inadequately treated previous infection, lack of compliance to therapy). Here we report a case of a 33-year-old Saudi female who had a significant travel history, and a peripheral blood smear (PBS) revealed mixed infection with *P. falciparum *and *P. vivax*. The case was successfully treated with a combination therapy of artemisinin and primaquine with follow up testing at three, seven, 14, and 28 days.

Mixed malaria infections are especially reported in travelers to endemic areas. Hence, adequate diagnosis and appropriate treatment of the cases contributes majorly to preventing relapse and controlling the disease. Travel consultations should be given to all travelers before their trips to endemic countries.

## Introduction

Malaria is a vector-borne disease caused by protozoan parasites belonging to genus *Plasmodium,* and is considered to be one of the most important parasitic infections throughout the world, resulting in high mortality rates ^1^. There were previously four known species responsible for human malaria: *P. falciparum*, *P. vivax*, *P. ovale*, *P. malariae*. Recently, it was discovered that there is also a fifth species, known as *P. knowlesi* ^2^. *P. falciparum* and *P. vivax* have the widest distribution, while *P. malariae* has a very low prevalence ^3^.

The Kingdom of Saudi Arabia (KSA) has reported 61 and 38 indigenous malaria cases in the years 2018 and 2019 respectively ( World Health Organization (WHO), World Malaria Report 2020). The indigenous cases in KSA have decreased drastically over the years (272 cases in 2016 to 38 in 2019) due to the implementation of the National Malaria Control Program (NMCP), the establishment of mobile units with specific focus on undocumented migrants from neighboring countries where diagnosis, treatment and medical care are provided free of charge to all imported cases ^4^ ( WHO, 2020).

The majority of malaria infection cases in KSA are due to *P. falciparum* (97%) followed by *P. vivax* (2%). *P. falciparum* causes malignant tertian malaria occasionally associated with severe complications in infected individuals. Another concern with *P. falciparum* infection is recrudescence, resulting in treatment failure and emergence of antimalarial drug resistance to chloroquine ( WHO, 2020).

## Case report

A 33-year-old Saudi female, Eastern region resident was admitted to the ‘Infectious Diseases’ ward of our teaching hospital King Fahad Hospital of the University, Imam AbdulRahman bin Faisal University with ‘fever of other and unknown origin’. The patient presented with a seven-day fever with chills and complained of decreased appetite and generalized body pain associated with dark urine, pale stool and diarrhea, with history of fever with chills and generalized body pains for one week. On taking detailed history, the patient stated that she had recently returned from a 20-day trip to the Ivory Coast. The patient didn’t have any form of comorbidities and denied any history of animal contact, immunocompromised status, or intake of any kind of prescribed or illicit drugs or raw milk ingestion during this period. Family history of the patient revealed absence of hereditary blood diseases and was of insignificant relation to the patient’s clinical presentation. The patient did not seek any medical facility prior her admission to our hospital and has only self-administrated paracetamol tablets in response to the fever and body aches.

On recording the vital signs (measurement of body temperature, weight, blood pressure and pulse rate), the patient was febrile with an oral temperature of 39.9°C, tachycardic with a pulse rate of 118 beats/minute, blood pressure of 135/85 mmHg, and respiratory rate of 20 breaths/minute.

Upon physical examination, the patient appeared ill and had pale conjunctivae. Lymphadenopathy was noted in the left axillary (1×1 cm and 0.5 × 1.5cm) and left inguinal region (0.5 × 2cm). Skin appeared normal and there were no petechiae. The results of hematological testing revealed the patient had normocytic normochromic anemia, pancytopenia ( Table 1), and liver function test (LFT) were borderline with significantly high levels of lactate dehydrogenase indicating an active haemolysis ( Table 2). The peripheral blood smear (PBS) depicted *P. falciparum* gametocytes (crescent) and *P. vivax* gametocytes (of ameboid shape with Schüffner's dots). PBS was then repeated on three, seven, 14, and 28 days. C-reactive protein (CRP) plasma level and erythrocyte sedimentation rate (ESR), which are both inflammatory blood markers that indicate an ongoing infection, were higher than the normal levels ( Table 3). Serological testing included measuring the antibody levels (both their Immunoglobulin (Ig) IgG and IgM forms) for Epstein–Barr virus (EBV), and Cytomegalovirus (CMV). The patient’s results were positive for both EBV IgG and CMV IgG ( Table 4). It is worth mentioning that there were no challenges encountered either in clinically examining the patient or getting access to the laboratory diagnostic tests. 

**Table 1.** Complete blood count (CBC), differential white blood cells (WBCs) count and coagulation tests.

Test	Patient result	Reference interval
Hemoglobin	7.6 g/dL	12–16 g/dL
Hematocrit	22.8 %	37–47 %
Red blood cells	2.65×10 ^12^/L	4.2–5.5×10 ^12^/L
MCV	85.9 fL	80–94 fL
MCH	28.5 pg	27–32 pg
WBCs	3.2×10 ^9^/L	4–11×10 ^9^/L
**Differential WBCs count**		
Neutrophils	41.7 %	40–75%
Lymphocytes	44.6 %	20–45%
Monocytes	11.9 %	3–9%
Eosinocytes	1.3 %	0×6 %
Basophils	0.5 %	0–1%
Platelets	120×10 ^9^/L	140–450×10 ^9^/L
**Coagulation tests**		
Prothrombin time	13.8 seconds	10–14 seconds
Partial thromboplastin time	29 seconds	30–45 seconds
International Normalized Ratio	1.3 ISI	1-2 ISI

**Table 2.** Biochemistry and liver function tests.

Test	Patient result	Reference interval
Total Protein	7.5 g/dL	6.4–8.2 g/dL
Albumin	3.0 g/dL	3.4–5.0 g/dL
Sodium	136 mEq/L	136–145 mEq/L
Potassium	4.0 mEq/L	3.5–5.1 mEq/L
Blood urea nitrogen	6 mg/dL	7–18 mg/dL
Creatinine	0.68 mg/dL	0.6–1.0 mg/dL
Lactate dehydrogenase	589 U/L	81–234 U/L
Total Bilirubin	0.7 mg /dL	0.2–1.0 mg /dL
Direct Bilirubin	0.19 mg/dL	0.05–0.2 mg/dL
Alkaline phosphatase	135 U/L	46–116 U/L
Gamma-glutamyl transferase	23 5–55 U/L	5–55 U/L
Aspartate aminotransferase	28 U/L	15–37 U/L
Alanine transaminase	30 U/L	14–36 U/L

**Table 3.** Inflammatory blood markers tests.

Test	Patient result	Reference interval
C-reactive protein quantitative test	7.00 mg/dl	0.05–0.3 mg/dl
Erythrocyte sedimentation rate	>50 mm/hour	0–20 mm/hour

**Table 4.** Virology screening tests.

Test	Patient result
Epstein-Bar virus (EBV) IgG IgM	Positive Negative
Cytomegalovirus (CMV) IgG IgM	Positive *Equivocal*

**Figure 1.**
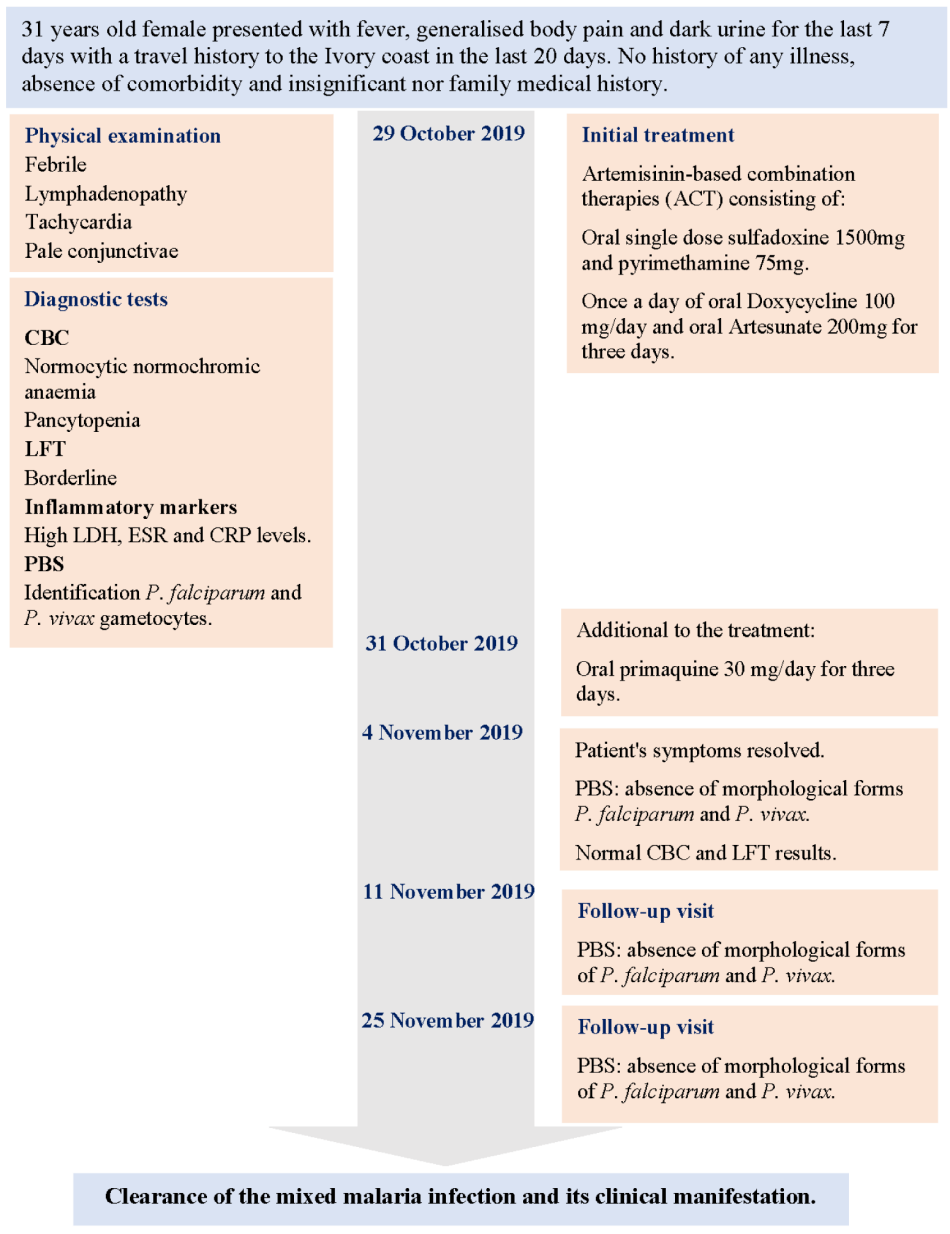
Case management timeline of *P. falciparum* and *P. vivax* mixed infection. CBC Complete blood count; LFT Liver function test; LDH Lactate Dehydrogenase; ESR Erythrocyte sedimentation rate; CRP C-reactive protein; PBS Peripheral blood smear.

The patient was treated as follows: oral doxycycline 100 mg once a day, artesunate 200mg orally once a day for three days, and sulfadoxine and pyrimethamine 1500mg + 75mg single dose orally on day one. On the third day, after excluding glucose-6-phophate dehydrogenase (G6PD) deficiency by measuring the G6PD enzyme level, oral primaquine 30 mg/day for three days was added to the treatment regimen. After seven days of the treatment, the patient's symptoms had resolved; the PBS was repeated and revealed the absence of morphological forms of both parasites along with normal CBC and LFT results. Treatment and care provided to the patient was free of any financial charges. During the treatment course the patient did not develop any adverse effects due to the administrated medications. Figure 1 summarizes the timeline of the case management course.

## Discussion

Several studies conducted worldwide and in KSA report that malaria mixed infections are not uncommon ^5– 8^. The majority of malaria studies in KSA were studies conducted in the Southwest area and included cases of mixed *P.falciparum* and *P.vivax* infections ^9– 11^. However, in the Eastern province of KSA where this case is reported, there have been few cases of single or mixed infection malaria reported due to effective control measures which resulted in the termination of malaria transmission ^11^.

Mixed infections in malaria can be attributed to one or more of four reasons: several species of *Anopheles* can carry more than one species of the parasite; *P.vivax* can remain as hypnozoites in the liver, which can be cause a relapse; recrudescence in *P.falciparum* infection due to drug resistant forms; and incomplete treatment of a previous infection ^12^.

Microscopic examination of PBS for the morphological forms of the malarial parasite remains the gold standard for laboratory confirmation of the infection ^11^. However, making a diagnosis of malaria mixed infection based on a PBS microscopic diagnosis is challenging. This difficulty can be due to: low levels of parasitemia; altered morphology of the parasite due to self-treatment; or the similarity in *Plasmodium* species, e.g. *P. knowlesi* is often misdiagnosed as *P. malariae* or *P. falciparum* ^12^.

Several tests can be used in conjunction with the PBS such as rapid diagnostic tests (RDT), antibody detection using indirect fluorescent antibody tests (IFA), and polymerase chain reaction (PCR) to identify the infection. However, all these methods have their own limitations. The RDT cannot detect low level parasitemia while IFA is time consuming, subjective, requires fluorescence microscopy and a trained observer ^13^. However, molecular tests such as PCR can assist in definitive species determination and also can detect low level parasitemia (about one parasite/μL of blood) ( Centers for Disease Control and Prevention) ^13^. It was not possible to perform a PCR test in the time this patient was diagnosed in our hospital due to the unavailability of the test in our laboratory, which was a limitation in the diagnostic approach.

The main strategy in treatment of uncomplicated malaria consists of oral therapy with a combination of two agents, the artemisinin and a partner drug that eliminates the remaining parasites, (in the case of chloroquine resistance) or chloroquine monotherapy (in the case of chloroquine sensitivity) ( WHO, 2020). Treatment for chloroquine-resistant *P. falciparum* should be administered in the setting of known exposure to a chloroquine-resistant region, unknown prevalence of chloroquine resistance, or uncertain exposure history. As a result, artemisinin-based combination therapies (ACT) have become the first line of treatment for *P. falciparum* and mixed malaria infections. The ACT regimens have few side effects and are cidal to all the asexual stages of the parasite in the blood, causing rapid clearance of the infection ^14^.

In this reported case, the patient received an ACT regimen comprising artesunate, sulfadoxine and pyrimethamine. Doxycycline was also chosen as it has high patient compliance and can be given once daily, and the seven-day course of these tablets finishes after the ACT regimen. Primaquine is given in malarial mixed infections along with ACT as it is the only drug recommended to eliminate the intrahepatic forms (hypnozoites) of *P. vivax* and gametocytes of *P. falciparum*. However, the patient’s G6PD levels should be tested to prevent primaquine-induced hemolysis as a phenolic metabolite, 5-hydroxyprimaquine (5-HPQ), mediates primaquine hemotoxicity by generating reactive oxygen species (ROS) within erythrocytes that overwhelm antioxidant defenses ^15, 16^. Supportive care should be given to the patient to prevent morbidity and mortality. In addition, inappropriate combinations and inadequate dosing of drugs should be avoided to prevent drug resistance and relapse ( WHO, 2021). As there is no available malarial vaccine, chemoprophylaxis can be administered to travelers before visiting the endemic areas according to ( WHO, 2021).

Overall, this reported case has one limitation at the diagnostic level: the unavailability of the quantitative real-time PCR (q-PCR) test to confirm the identification of both the malaria species and to measure the level of the parasitemia, since q-PCR is more reliable in detecting mixed infection at levels of low parasitemia ^9^. Although it would have been an added value in terms of confirmation, q-PCR unavailability did not affect the proper diagnosis. The accuracy in the PBS examination enabled the identification of both malaria species which is considered one of the strengths of this case. Another strength of this reported case is the timely, appropriate management plan. The patient responded well with the treatment without any side effects and the parasitemia was resolved in due time.

To conclude, malarial infection can be controlled with different measures taken at different levels: appropriate vector control measures; early case detection; timely management; entomological monitoring; population movement monitoring and surveillance; reporting of cases; and public health education strategies.

## Data availability

All data underlying the results are available as part of the article and no additional source data are required.

## Consent

Written informed consent for publication of patient’s clinical details was obtained from the patient.
