# Supplementary material for: Case Report: Case report: Mixed infection of Plasmodium vivax and Plasmodium falciparum in a tertiary hospital
Source: F1000Res. 2021 Aug 9;10:779. [Version 1] doi: 10.12688/f1000research.53162.1 (PMC8456375; doi:10.12688/f1000research.53162.1)
Supplement: Peer review history

# Peer review: Reviewer response for version 1

**DOI:** 10.5256/f1000research.56516.r92549

**Published:** 2021-09-21

**Authors:** Ali M. Gabali

**Affiliations:** 1 Department of Pathology, Karmanos Cancer Center, Wayne State University School of Medicine, Detroit, MI, USA

`The paper reviewed presented a Mixed infection of *Plasmodium vivax* and *Plasmodium falciparum* in a tertiary hospital. The authors discussed thoroughly the clinical presentation of concomitant *Plasmodium vivax* and *falciparum* infection in this case and the clinical course and modality of therapy used. The case is addressing an important issue; the presentation and clinical course are largely well explained and appropriately used and the paper is well written. My conclusion is to approve the paper for indexing.

Are enough details provided of any physical examination and diagnostic tests, treatment given and outcomes?

Yes

Is the case presented with sufficient detail to be useful for other practitioners?

Yes

Is sufficient discussion included of the importance of the findings and their relevance to future understanding of disease processes, diagnosis or treatment?

Yes

Is the background of the case’s history and progression described in sufficient detail?

Yes

Reviewer Expertise:

Immunology, hematology and hematopathology

I confirm that I have read this submission and believe that I have an appropriate level of expertise to confirm that it is of an acceptable scientific standard.

# Peer review: Reviewer response for version 1

**DOI:** 10.5256/f1000research.56516.r91522

**Published:** 2021-09-01

**Authors:** Mira El Chaar

**Affiliations:** 1 Faculty of Health Sciences, University of Balamand, Beirut, Lebanon

The case study presented by the authors is well elaborated. The authors describe in detail the sequence of the clinical outcome of a patient who was admitted to the hospital with *plasmodium* species coinfections.

 The Sequence of the case is well presented including the background, the method of detection, the result, and the discussion.

 The authors have tackled the limitation of this study in particular the absence of real-time PCR to study the parasitaemia level in the patient which is very important.

 The article needs minor changes for indexing including the following:

1. All laboratory tests and markers could be presented in one table instead of 4 tables. If the authors prefer to keep it as such, table 4 should be deleted.
2. The authors should mention the year when the patient was admitted to the hospital in the result part, although it is included in figure 1.
3. It is worth mentioning in the discussion about the prevalence of coinfection of *plasmodium* species at the regional and international levels.
4. The authors should mention in the discussion if coinfection can increase the severity of the disease.

Are enough details provided of any physical examination and diagnostic tests, treatment given and outcomes?

Yes

Is the case presented with sufficient detail to be useful for other practitioners?

Yes

Is sufficient discussion included of the importance of the findings and their relevance to future understanding of disease processes, diagnosis or treatment?

Yes

Is the background of the case’s history and progression described in sufficient detail?

Yes

Reviewer Expertise:

NA

I confirm that I have read this submission and believe that I have an appropriate level of expertise to confirm that it is of an acceptable scientific standard.
